# In-service training programme for health and social care workers in the Philippines to strengthen interprofessional collaboration in caring for older adults: a mixed-methods study

**DOI:** 10.1186/s12961-022-00914-2

**Published:** 2022-11-29

**Authors:** Keiko Nakamura, Kathryn Lizbeth L. Siongco, TJ Robinson T. Moncatar, Lourdes Marie S. Tejero, Shelley Ann F. De La Vega, Sheila R. Bonito, Richard Javier, Takako Tsutsui, Tran Dai Tri Han, Man Thi Hue Vo, Yuri Tashiro, Saber Al-Sobaihi, Kaoruko Seino, Thang Van Vo, Fely Marilyn E. Lorenzo, Carmelita C. Canila

**Affiliations:** 1 Department of Global Health Entrepreneurship, Tokyo Medical and Dental University, Yushima 1-5-45, Bunkyo-ku, Tokyo, 113-8519 Japan; 2 WHO Collaborating Centre for Healthy Cities and Urban Policy Research, Tokyo, Japan; 3 College of Nursing, University of the Philippines Manila, Manila, Philippines; 4 Department of Health Policy and Administration, College of Public Health, University of the Philippines Manila, Manila, Philippines; 5 Technology Transfer and Business Development Office, University of the Philippines Manila, Manila, Philippines; 6 Institute on Aging, National Institutes of Health, University of the Philippines Manila, Manila, Philippines; 7 Human Resource Development Office, University of the Philippines Manila, Manila, Philippines; 8 University of Hyogo, Kobe, Japan; 9 University of Medicine and Pharmacy, Hue University, Hue, Viet Nam; 10 ASEAN Institute for Health Development, Mahidol University, Salaya, Thailand; 11 Commission on Higher Education of the Philippines, Manila, Philippines

**Keywords:** Interprofessional collaboration, In-service training, Geriatric care, Attitudes towards health care teams

## Abstract

**Background:**

A growing number of older adults require complex care, but coordination among professionals to provide comprehensive and high-quality care is perceived to be inadequate. Opportunities to gain the knowledge and skills important for interprofessional collaboration in the context of geriatric care are limited, particularly for those already in the workforce. A short-term training programme in interprofessional collaboration for health and social care workers in the Philippines was designed and pilot tested. The programme was devised following a review of the literature about geriatric care education and group interviews about training needs. The objectives of this paper are to introduce the training programme and to evaluate its influence on attitudes and readiness to collaborate among participants using both quantitative and qualitative methodologies.

**Methods:**

A total of 42 community health workers and 40 health institution workers participated in the training in July 2019. Quantitative indicators were used to evaluate attitudes towards and readiness for collaboration before and after the training. Content analysis was performed of responses to open-ended questions asking participants to evaluate the training. A convergent parallel mixed-methods design was applied to determine the patterns of similarities or differences between the quantitative and qualitative data.

**Results:**

Significant improvements were seen in scores on the Attitudes Towards Health Care Teams Scale among community health (*P* < 0.001) and health institution (*P* < 0.001) staff after the training. Scenario-based case studies allowed participants to work in groups to practise collaboration across professional and institutional boundaries; the case studies fostered greater collaboration and continuity of care. Exposure to other professionals during the training led to a deeper understanding of current practices among health and social care workers. Use of the scenario-based case studies followed by task-based discussion in groups was successful in engaging care professionals to provide patient-centred care.

**Conclusions:**

This pilot test of in-service training in interprofessional collaboration in geriatric care improved community and health institution workers’ attitudes towards such collaboration. A 3-day training attended by health and social care workers from diverse healthcare settings resulted in recommendations to enhance collaboration when caring for older adults in their current work settings.

**Supplementary Information:**

The online version contains supplementary material available at 10.1186/s12961-022-00914-2.

## Background

The need for geriatric care is increasing rapidly in the Asia–Pacific region [1]. Considering the complex health and social care needs of older adults, it is critical to organize professionals from different disciplines to plan, coordinate and deliver care across all levels of the health system [2].

In most of the Asia–Pacific region, geriatric care is now included in the undergraduate curriculum at medical, nursing and allied health schools, and it is backed by policies to support older people’s right to health [3]. However, the number of healthcare workers remains inadequate, and the workforce is ill-equipped to provide appropriate care for older adults. It is especially concerning that the existing health care workforce—including those currently serving in leadership roles in communities, hospitals and institutions—lacks exposure to contemporary education and training in geriatric care. Moreover, training in collaboration across professional disciplines is not part of the formal education and training of healthcare professionals.

Interprofessional collaboration (IPC) will play a vital role in easing the negative effects of fragmented health systems, including the constraints imposed by a dearth of healthcare professionals. IPC enables different health and social care professionals to work together to render the highest-quality care to improve health outcomes [4]. IPC in the primary healthcare setting is defined as integrated cooperation among health professionals from diverse professional backgrounds, working together to share their skills and competencies, allowing for the effective use of human resources for patient care. IPC training for the workforce that currently provides geriatric care should be developed to meet rapidly growing needs.

Building on existing academic partnerships and expertise, formative research was conducted in the Philippines and Viet Nam that included 70 focus group interviews with 348 health and social care professionals. A common theme that emerged from both countries was that a growing number of older adults require complex care for geriatric syndromes and noncommunicable diseases, but comprehensive, equitable and high-quality approaches to care are perceived to be inadequate and non-holistic, largely due to separation between the health and social welfare sectors [5]. Interviews showed that IPC practices are generally considered to be administrative in both the Philippines and Viet Nam [5].

A short-term, IPC training programme was designed and pilot tested with groups of health and social care workers involved in geriatric care in communities, hospitals and other care institutions in the Philippines. The objectives of this paper are to introduce the training programme and to evaluate its influence on attitudes towards collaboration and readiness to collaborate among participants using quantitative and qualitative methodologies. The mixed method combines elements of quantitative and qualitative methods in order to answer the research question. Of the three main types of the mixed method approaches—exploratory sequential, explanatory sequential and convergent designs—this study uses the convergent design which involves quantitative and qualitative data collection and analysis at similar times, followed by an integrated analysis [6]. We chose this method because some of the expected outcomes of the intervention are measured quantitatively using standardized scales and metrics, while other outcomes are not quantifiable and are better captured qualitatively. By merging the quantitative and qualitative results, a more complete understanding could be gained about the complexity of the training outcomes than could be gained by either quantitative or qualitative results alone. It was hoped that exposure to professionals from other disciplines during training would lead health and social care workers to develop a deeper understanding of care practices and available services.

## Methods

### Training programme and modules

A team of experts in public health, gerontology, nursing and interprofessional education (IPE) developed a competency-based, 3-day pilot programme and consulted with central and local government agencies in the Philippines to finalize the programme and the protocol for a pilot test.

Table 1 provides more in-depth information about the 10 training modules, which included an overview of aging, health conditions in older age, effective communication while caring for older adults, IPC, geriatric syndromes, comprehensive geriatric assessment (CGA), interprofessional group work, care management and coordination, community support for older adults and enhancing IPC in the participant’s current setting.

**Table 1:** Modules in the 3-day pilot training in IPC in geriatric care, Philippines, 2019

Day	Session	Module	Topics	Competencies	Activities
1	AM	I. Overview of healthy ageing	a. Healthy ageing b. Factors influencing health in older age	1. Gain knowledge regarding functional ability in older age that enables well-being 2. Identify health-related changes in older age 3. Determine factors that influence health in older age	Seminar-based discussion Interview of patients by instructor
		II. Health conditions in older age	a. Impact of health-related changes on intrinsic capacity	1. Develop understanding of changes in movement, sensory and cognitive functions, and age-associated conditions 2. Develop understanding of the impact of health-related changes on the intrinsic capacity of older persons 3. Identify evidence-based recommendations and management of common age-related changes and conditions among older persons	Seminar-based discussion Case study Role play
	PM	III. Effective communication in caring for older adults	a. Verbal and nonverbal communication techniques b. Issues in communicating with older adults c. Interprofessional collaborative competencies: c. 1. Communication c. 1. a. Listening c. 1. b. Sharing information c. 1. c. Giving and receiving feedback	1. Identify effective communication techniques to use while providing care for older adults 2. Determine special communication needs according to the older adult’s capacity and limitations 3. Demonstrate strategies for communicating with older persons 4. Identify various methods of providing clinical information to nurses, family members and other members of the healthcare team	Seminar-based discussion Case study Video presentation
		IV. IPC	a. Concept of interprofessional education and collaborative practice b. Roles, responsibilities and scope of practice c. Interprofessional collaborative competencies: c. 1. Values and ethics c. 1. a. Creativity/innovation c. 1. b. Professional relationships c. 2. Communication c. 2. a. Listening c. 2. b. Sharing information c. 2. c. Giving and receiving feedback c. 3. Collaboration c. 3. a. Roles, responsibilities, scope of practice c. 3. b. Decision-making and problem-solving c. 3. c. Self-reflection c. 3. d. Personal contribution d. Interprofessional education and collaborative practice in the workplace	1. Develop understanding of IPC competencies and their practical application to the care of older adults 2. Identify the roles, responsibilities and scope of practice of each team member 3. Develop strategies for incorporating IPC in the workplace	Seminar-based discussion Group work
**Lessons learned sessions**					Reflection with short quizzes and games
2	AM	V. Geriatric syndromes	a. Dementia b. Falls c. Polypharmacy	1. Assess older adults for presence of geriatric syndromes a. Risk factors b. Assessment skills c. Tools 2. Identify preventive and curative interventions corresponding to each syndrome a. Screening and referral process b. Interprofessional management	Seminar-based discussion
		VI. Comprehensive geriatric assessment	a. Assessment of physical, cognitive, psychological, social and functional status of older adults (dementia, falls and polypharmacy) b. IPC in comprehensive geriatric assessments	1. Define the comprehensive geriatric assessment 2. Identify components of the comprehensive geriatric assessment 3. Describe the process of conducting a comprehensive geriatric assessment 4. Identify the benefits of involving an interprofessional team to conduct a successful assessment	Seminar-based discussion Demonstration of comprehensive geriatric assessment
	PM	VII. Interprofessional group work	a. Depression b. Functional decline (stroke case study) c. Care coordination d. IPC	1. Identify health and social concerns 2. Identify prevention strategies and interventions relevant to the case 3. Perform a comprehensive geriatric assessment 4. Conduct discussion with the geriatric care team and develop a care plan (treatment, referral, follow-up)	Case scenario Group work presentation
**Lessons learned sessions**					Reflection with short quizzes and games
3	AM	VIII. Care management and coordination	a. Self-awareness b. Interpersonal skills c. Teamwork mechanisms	1. Gain self-awareness 2. Integrate experiences of health and social care professions to improve decisions about health and care 3. Engage self and others to constructively manage disagreements	Seminar-based discussion Reflection
		IX. Community support for older adults	a. Integrated care for older adults b. Community policies, programmes, resources c. Safe and well-coordinated care d. Caregiver support	1. Discuss the issues and principles of providing integrated care to older adults 2. List the community policies, programmes and resources available to meet the healthcare needs of older adults 3. Map safe and well-coordinated care to be delivered during transitions across healthcare settings that involve healthcare professionals, the family and informal caregivers 4. Describe issues that arise and interventions needed to provide caregiver support	Seminar-based discussion Video presentation
	PM	X Enhancing IPC in the current setting	a. IPC in practice	1. Identify any anticipated changes to practices (individual, team at barangay, teams at hospital, institution or other organization) 2. Identify partners to enhance IPC in geriatric care 3. Identify practical steps to enhance IPC with partners	Small-group and large-group discussions
**Lessons learned sessions**					Reflection with short quizzes and games

The pedagogy of IPE followed in the training programme was focused on setting clear and attainable outcomes to the participants, engaging them in active learning, providing reflective opportunities and delivering timely feedback during the course of training [7]. To emphasize the centrality of group work in IPE, adult learning principles were employed to enable a higher sense of self-direction and to draw upon the knowledge and experiences of the participants to facilitate learning and attainment of the expected outcomes. Multiple teaching and learning methodologies were applied to organize individual classes and sessions about the lessons learned, including seminar-based discussions, interviews, use of the CGA tool to analyse the patients in the case studies, presentation of case studies, role plays, video presentations, group work, reflection and small-group and large-group discussions that prompted interaction and active participation between team members. Before participants used the CGA tool in case studies, experts demonstrated its use with example cases.

The case-study group work required participants to collaborate to construct care plans in response to specific case scenarios. Box 1 shows the facilitator’s guide for the case studies. Examples of the case studies are provided in the Additional file 1: Appendix. Each case-study group consisted of eight to nine participants from different professional backgrounds. Participants discussed the scenario, listed their problems or concerns, proposed what action the team should take next and applied the CGA tool. The shared learning experience in the training programme can improve interprofessional communication skills by fostering mutual respect and engagement in shared decision-making among the participants from different professional backgrounds.

**Box 1.**
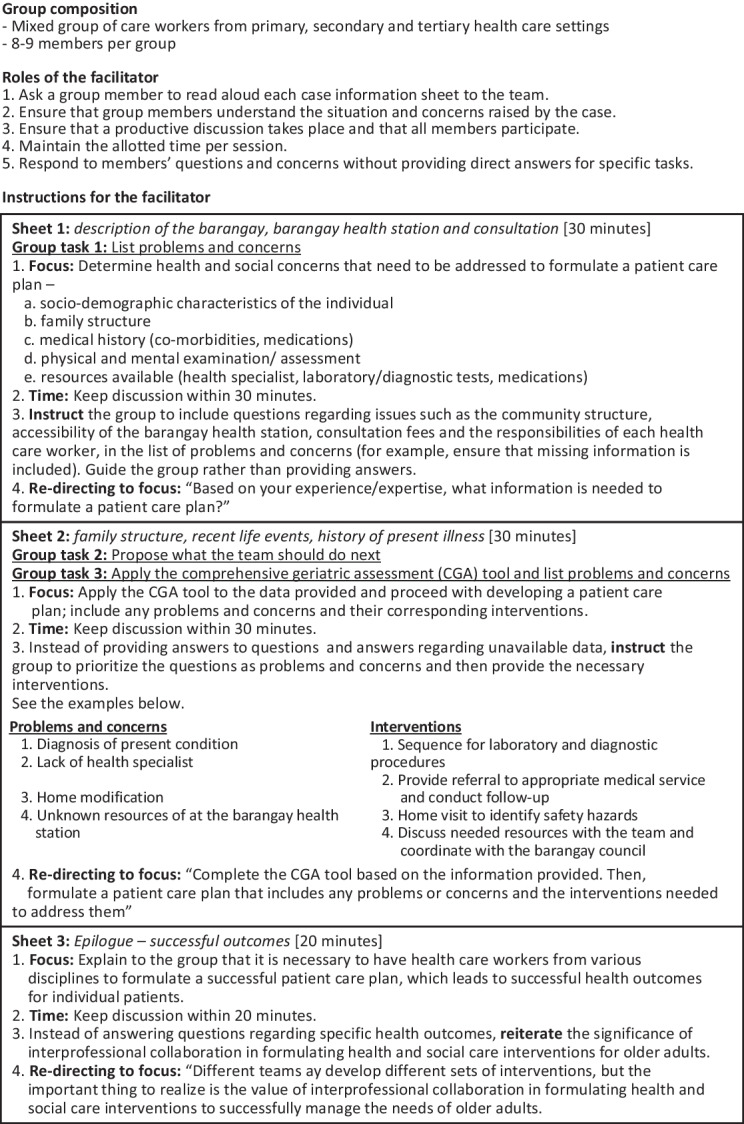
Facilitator’s guide for case-study group work

Evaluation of the training programme was based on the Kirkpatrick outcome model [8] which has been widely used as the framework for evaluation of IPE [9]. This model evaluates learner outcomes of educational initiatives, conceptualized as a hierarchy of outcomes, from measuring changes in reactions, attitudes and perceptions, and further moving towards organizational change. For this training programme, reactions, modification of attitudes/perceptions, acquisition of knowledge and skills, and behavioural change were evaluated through the use of standardized assessment tools. These outcomes can help explain the learner’s views of the learning experience, changes in reciprocal attitudes or perceptions, knowledge and skills development linked to IPC, and the transfer of interprofessional learning to their practice setting.

### Pilot training session

Participation in the pilot training session was voluntary. Altogether, 42 community health workers and 40 health institution workers were selected to participate. Health or social care workers who had been employed in the city or a local health institution (such as a hospital or nursing home) for at least 1 year and who had daily contact with older adults were eligible for inclusion. People with the following backgrounds were invited to participate: community health workers, nurses, nutritionist, physicians and rehabilitation therapists. Sample size calculation was derived from the expected difference between pre-test and post-test evaluation utilizing the Readiness for Interprofessional Learning Scale [10], with a computed dropout rate of 15%. Both the quantitative and qualitative data collections were performed on the same sample.

The pilot training session was conducted in July 2019 in Marikina and Tagaytay, cities in the Philippines with populations of 450 741 and 71 181, respectively [11]. Both cities had experience developing health promotion programmes focusing on IPC with the research team. The percentage of older adults aged ≥ 60 years in Marikina was 7.8%, and in Tagaytay was 6.9% [5]*.* These cities are composed of barangays (i.e. communities) that are the basic political and administrative unit in the Philippines and that serve as the primary channel for planning or implementing enhanced delivery of government programmes [12]*.*

### Quantitative evaluation

The outcome evaluation used a pre- and post-test design. Standardized self-administered questionnaires were used to measure attitudes towards and readiness for activities related to IPC. Three scales that had been translated into the Filipino language were administered: the Attitudes Toward Health Care Teams Scale (ATHCTS) [13], Readiness for Interprofessional Learning Scale (RIPLS) [14] and the Coordinated Activities Evaluation Scale (CAES) [15]. Details of these three scales are described elsewhere [16].

Changes in mean individual scores before and after the training were examined by paired *t*-test separately for community health and health institution workers. Statistical analyses were performed using SPSS version 25 (IBM, Armonk, NY, USA). Statistical significance was set at *P* < 0.05.

### Qualitative evaluation

A training programme evaluation questionnaire was developed to gain insights into the effectiveness of the programme in achieving its objectives, the satisfaction of participants with the programme and ways to improve it for future implementation. Participants described their experiences and the lessons learned by responding to open-ended questions: What did you like most about this training? How do you hope to change your practices as a result of this training? What new information did you learn from the topics presented?

All data were transcribed verbatim and translated into English. After transcription, an inductive content analysis using NVivo 12® (QSR International, Burlington, MA, USA) was conducted to initially determine the relevant descriptive codes. The identified codes were then sorted according to their commonalities and relationships to establish the emerging categories. Discussion among the authors subsequently followed to distinguish and agree on the overarching themes and the basis for the developed categories to ensure the accuracy of the qualitative findings [17, 18].

### Mixed-method analysis

**Fig. 1:**
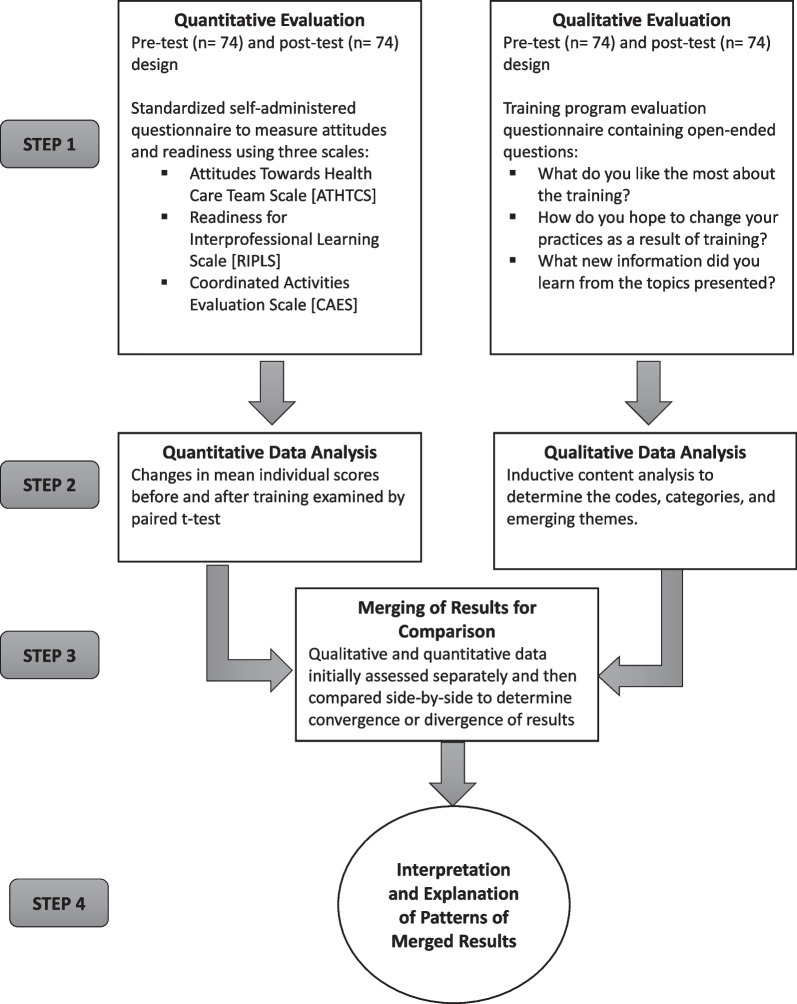
Diagram of parallel convergent mixed methods approach

After separate data collection and analysis of quantitative and qualitative information, a convergent parallel mixed-method analytical design was performed. A side-by side comparison of both sources of information was conducted in order to determine areas of convergence or divergence [6]. An iterative consultation and discussion within the research team was carried out to determine and validate the findings. Figure 1 illustrates the mixed-methods approach utilized in this study.

### Ethics approval

The study was approved by the WHO Research Ethics Review Committee (protocol no. ERC.003093), the Tokyo Medical and Dental University Ethics Review Board (approval no. M2017-232) and the Single Joint Research Ethics Board, Department of Health, Philippines (approval no. SJRED-2018-21). Written informed consent was obtained from all participants involved in the study before data were collected.

## Results

### Quantitative assessment

Of the 42 community health workers and 40 health institution workers who participated in the training, 39 community health workers and 35 health institution workers completed three evaluation scales both before and after training. Table 2 shows the mean and standard deviation (SD) of scores before and after training on the ATHCTS, RIPLS and CAES.

**Table 2:** Scores on the ATHCTS, the RIPLS and the CAES before and after in-service training on IPC, Philippines, 2019

Scale (score range)	Mean (SD) scores with *P* values^a^			
	Community health workers^b^ (*n* = 39)		Health institution workers^b^ (*n* = 35)	
ATHCTS (possible range 21–126)				
Before training	78.6 ± 6.3		89.1 ± 10.3	
After training	85.4 ± 5.5	< 0.001	98.7 ± 11.8	< 0.001
RIPLS (possible range 19–95)				
Before training	79.7 ± 10.0		85.0 ± 7.9	
After training	83.2 ± 7.3	0.037	87.4 ± 5.5	0.045
CAES (possible range 0–45)				
Before training	30.5 ± 7.7		25.6 ± 10.4	
After training	32.4 ± 8.5	0.191	30.4 ± 9.0	< 0.001

ATHCTS: Attitudes Toward Health Care Teams Scale; CAES: Coordinated Activities Evaluation Scale; RIPLS: Readiness for Interprofessional Learning Scale; SD: standard deviation

^a^*P* calculated using paired *t*-test

^b^Of the 42 community health workers and 40 health institution workers who participated, 39 community health workers and 35 health institution workers completed questionnaires both before and after training

Significant improvements in ATHCTS scores [18] were observed among community health workers (mean [SD] 78.6 [6.3] before training and 85.4 [5.5] after training out of a possible 126; *P* < 0.001) and health institution workers (89.1 [10.3] before training and 98.7 [11.8] after training; *P* < 0.001). RIPLS scores [19] also increased among both community health workers (79.7 [10.0] before training and 83.2 [7.3] after training out of a possible 95; *P* = 0.037) and health institution workers (85.0 [7.9] before training and 87.4 [5.5] after training; *P* = 0.045). CAES scores [20] significantly increased among health institution workers (25.6 [10.4] before training and 30.4 [9.0] after training out of a possible 45; *P* < 0.001), but it did not change significantly among community health workers. Thus, the training programme has great potential to enhance community health and health institution workers’ capacity for IPC.

### Qualitative data analysis

Table 3 presents the key themes derived by analysing participants’ responses to the open-ended questions in the programme evaluation. According to the participants, the factors that contributed to the successful implementation of the training programme included the competency of the trainers in delivering the topics in a way that could be easily understood by the participants, the provision of active learning experiences and the opportunity to cooperate with other health and social care professionals during the group work. The perceived success of the training programme was evident in the satisfaction expressed by the participants.

*I appreciated the knowledge I acquired from this training, and especially the group work with my colleagues, nurses and doctors. All of us spoke and learned from each other’s opinions about how to improve the patient’s condition. In my opinion, our group was successful in achieving good outcomes for the patient because we listened to each other.* [Community health worker]

*All topics that were discussed and taught allowed me to gain useful knowledge that I could use to improve my practice in the workplace.* [Community health worker]

**Table 3:** Content analysis of participants’ experiences and lessons learned from the in-service pilot training programme on IPC, Philippines, 2019

Themes	Categories	Responses (*N* = 74)
Factors for successful implementation of the training programme	Competency of facilitators	7
	Active learning experience	3
	Interprofessional group work	3
	Participant satisfaction	12
	Information dissemination	1
Benefits of the training programme	Self-awareness	29
	Understanding the role of each member in an IPC team	5
	Knowledge gained	13
	Practical application to the workplace	6
Factors facilitating IPC in the primary healthcare setting	Attitude change	7
	Patient-centred care for older adults	4
	Awareness of the setting	2
	Identify strengths and weaknesses	3
	Understand benefits of IPC in providing patient care	7
	Goal planning	1
The key lessons learned from the training	Care for older adults	6
	IPC	13
	Community support for older adults	6
	Comprehensive geriatric assessment	4
	Geriatric syndromes	15
	Health trajectories in older age	2
Interprofessional competencies for community health workers	Coordination by team leader	1
	Communication competency	8
	Collaboration	16
	Accountability	1
	Home visits	2
	Referrals	1
	Compassion in caring for older adults	8
	Care programme implementation	1
	Problem solving	3
	Interagency collaboration	2

This study also identified other benefits of the programme, as reported by the participants afterwards. Benefits in self-awareness were mentioned by 29 participants, and knowledge gain was mentioned by 13; other benefits included understanding the role of each member on an IPC team and the applicability of the training content to the workplace.

*First and foremost, the programme helped me to learn more about myself and think about my purpose and what I can contribute to achieving success as a group.* – Community health worker

*I learned to better know myself and my capabilities as a member of an IPC team. All the topics were useful to advance my knowledge of caring for older adults. This is important to help improve my skills in serving the community.* – Community health worker

As the key lessons learned from the training, 15 participants mentioned geriatric syndromes, and 13 mentioned learning how to work as part of an IPC team. Other key lessons identified included how to care for older adults, knowledge about the community support available for older adults, the CGA and health trajectories in older age. Furthermore, the IPC competencies required by community health workers were identified as communication, collaboration, accountability and compassion. Factors that supported or facilitated the practice of IPC in the primary healthcare setting were identified as attitude change, knowledge about patient-centred care, how to identify the strengths and weaknesses of care providers, and the benefits of IPC in patient care.

*What I would like to improve in my attitude is to be open-minded towards the situation of other people and of my team, to understand the circumstances that affect the patients [in the case study] and to be united to collaborate for the benefit of others and practice equality in healthcare provision by accepting all patients regardless of their need and available resources, so that we could extend help to all and especially to vulnerable groups.* – Community care midwife

*We would be able to easily provide solutions to problems in the workplace through interprofessional collaboration and coordination of the entire team.* – Community health nurse

*Topics included are focused on how to care for older adults and how to respond to their needs, such as caring for those with geriatric syndromes and knowing what community support is available for older adults, and how to properly treat health conditions in older adults and provide solutions for their problems.* – Health institution caregiver

### Integrated results

When analysed together, the quantitative and qualitative results converged to indicate the positive engagement of care professionals in providing collaborative geriatric care as a result of their exposure to the in-service IPC training programme. In the quantitative analysis, significant improvement in the mean and SD scores for ATHCTS, RIPLS and CAES was observed after the training compared to baseline among participating care professionals from both the community and health institutions. The qualitative content analysis also showed that the opportunity to cooperate and actively learn with other care professionals during the training programme resulted in a deeper understanding of the value of IPC, a positive change in attitude towards IPC, increased knowledge about IPC-based care and the roles of various providers, increased readiness to apply their learning towards effective geriatric care delivery, and improvement in how to work and coordinate as a team.

## Discussion

A short-term, competency-based, in-service training programme on IPC in geriatric care improved the attitudes of community health and health institution workers towards IPC. Exposure to other professionals during the training led to health and social care workers having a deeper understanding of current practices. Using scenario-based case studies for group work, followed by task-based discussion, showed success in encouraging care professionals to play a vital role in providing patient-centred care.

### Differences in scores before and after training

Before training, scores on both the ATHCTS and the RIPLS were high among health institution workers compared with those among community health workers. The differences could be due to experiences working with various professionals and participating in continuing education and conferences related to geriatric care. Health institution workers in geriatric care may have gained IPC skills through informal communication with allied health professionals in their workplace [19].

### Learning process

Critical to the learning process was the use of interactive approaches, such as role plays, case studies, group work, reflection, discussions, simulations, short quizzes, games and video presentations. The use of the local language, humour and contextualizing the lectures in the settings of the participants contributed to the assimilation of the concepts. Demonstrations by experts followed by demonstrations by participants helped reinforce the acquisition of skills and retention of knowledge. Having a practicum with an actual patient would have been ideal.

The case study group work included the presentation of individual scenarios that began with descriptions of the geographical characteristics of and the health system in the community; this was followed by a scenario describing a medical consultation at the community health centre and additional information about the patient’s family, recent life events, history of illness and the health services received. The groups that worked together on the case studies included community health workers, nurses, nutritionists, physicians and rehabilitation therapists from either community health or health institution settings, working together to use the CGA tool, enumerate problems and concerns and propose the team’s next steps. The discussion about the diagnosis, treatment plans, community referral and rehabilitation options was guided by a facilitator and allowed for information and experiences to be shared [20], comprehensive care options to be planned, care efficiency and continuity to be developed and coordination of care [21, 22].

The results of the qualitative data analysis provided further insight into the factors that contributed to the successful implementation of the programme, those that support the practice of IPC in the community health setting and the benefits of participation. The findings indicate that IPC is necessary and must be encouraged, in accordance with the definition of interprofessional collaborative practice in the primary healthcare setting [23].

Having an active learning experience and being able to express ideas and make suggestions were also identified as being helpful in achieving the objectives of the programme. Multiple teaching and learning strategies in the classroom, laboratory or in practice settings have been reported to facilitate attitudes favourable to the development of IPC [24]. Likewise, the participants in this study reported that the benefits gained from the programme included knowing oneself and understanding the role of each team member. A shared understanding of roles, values and goals among team members is essential as these are important components of interprofessional teamwork [25] that aims to improve access to care and patients’ outcomes and to reduce health disparities.

## Convergence of quantitative and qualitative assessment

The quantitative improvements in the participants’ attitude, readiness and practise of interprofessional teamwork and coordination were further validated by the qualitative data obtained from the training participants. A positive change in their attitude and perspective towards IPC and increased readiness to practise collaborative geriatric care emerged as key themes. These findings support the value of adopting and implementing an in-service training programme on IPC as a way to strengthen workforce capacity to deliver quality care for older adults at the primary care and institutional levels.

### Benefits of group work

Interprofessional group work is a highlight of the training programme. Participants with different professional backgrounds and years of experience formed groups that cut across all levels of the health system as they worked together on a case study. The group work allowed them to learn about different settings and exercise IPC across not just professional boundaries but also across institutional boundaries. These practices foster collaboration to ensure continuity of care for older adults, which is a challenge under current practices.

### Recommendations for improving collaboration in the workplace

On the last day, participants discussed in groups how IPC could be enhanced to benefit the care of older adults in their current workplace by reflecting on the training. Some of the recommendations they made included building a supportive organizational structure, for example, by holding interprofessional monthly meetings for health and social care workers; collecting and integrating information about the social and health needs of older adults; formalizing the use of standardized assessment tools for older adults; formalizing an integrated health and social services referral protocol; securing a mandate or support from local representatives, such as the mayor’s office, a city health officer or the medical director of a hospital or nursing home; and developing and monitoring a database of geriatric assessments that will be shared in individual primary care settings.

## Conclusions

IPC is practiced in the Philippines and Viet Nam, but it is generally considered to be an administrative function. Offering a short-term, competency-based, in-service training programme about IPC that focuses on geriatric care seems to be a promising way to improve the attitudes of community health and health institution workers towards the creation of interprofessional healthcare teams. Ensuring that the training includes a mix of health and social care workers from diverse settings is key to achieving learning outcomes and fostering improvements in continuity of care. Active learning can be promoted using scenario-based case studies that are followed by task-based discussions to develop an integrated care plan for older patients. Also essential are the existence of a strong partnership of stakeholders, well-trained staff, collaborative programme development and prioritization of the well-being of older adults by local governments.

## Supplementary Information

**Additional file 1: Appendix 1.** Case studies for training programmes to strengthen interprofessional collaboration in caring for older adults.

## Data Availability

The data presented in this study are not publicly available as the data contain potentially sensitive and personally identifiable data. However, the data are available upon request from the corresponding author.

## Abbreviations

ATHCTS: Attitudes Toward Health Care Teams Scale
CAES: Coordinated Activities Evaluation Scale
CGA: Comprehensive geriatric assessment
IPC: Interprofessional collaboration
RIPLS: Readiness for Interprofessional Learning Scale
SD: Standard deviation

## References

[1] United Nations Population Fund Asia Pacific. Perspectives on population ageing in the Asia-Pacific Region. Bangkok (Thailand): United Nations Population Fund; 2017. https://asiapacific.unfpa.org/en/publications/perspectives-population-ageing-asia-pacific-region. Accessed 5 Nov 2021.

[2] Ploeg J, Markle-Reid M, Fisher A, Bookey-Bassett S, Chambers T, Kennedy L (2017). An exploration of experts’ perceptions on the use of interprofessional education to support collaborative practice in the care of community-living older adults. J Interprof Care.

[3] Williamson C. Policy mapping on ageing in Asia and the Pacific: analytical report. Chiang Mai (Thailand): HelpAge International; 2015. http://www.refworld.org/pdfid/55c9e6664.pdf. Accessed 5 Nov 2021.

[4] World Health Organization. Framework for action on interprofessional education and collaborative practice. Geneva: World Health Organization; 2010. https://apps.who.int/iris/handle/10665/70185. Accessed 13 Dec 2021.

[5] Moncatar TRT, Nakamura K, Siongco KLL, Seino K, Carlson L, Canila CC (2021). Interprofessional collaboration and barriers among health and social workers caring for older adults: a Philippine case study. Hum Resour Health.

[6] Creswell JW, Creswell JD (2018). Research design: qualitative, quantitative, and mixed methods approaches.

[7] Chan LK, Ganotice F, Wong FKY, Lau CS, Bridges SM, Chan CHY (2017). Implementation of an interprofessional team-based learning program involving seven undergraduate health and social care programs from two universities, and students’ evaluation of their readiness for interprofessional learning. BMC Med Educ.

[8] Gillan C, Lovrics E, Halpern E, Wiljer D, Harnett N (2011). The evaluation of learner outcomes in interprofessional continuing education: a literature review and an analysis of survey instruments. Med Teach.

[9] Danielson J, Willgerodt M (2018). Building a theoretically grounded curricular framework for successful interprofessional education. Am J Pharm Educ.

[10] Wang R, Shi N, Bai J, Zheng Y, Zhao Y (2015). Implementation and evaluation of an interprofessional simulation-based education program for undergraduate nursing students in operating room nursing education: a randomized controlled trial. BMC Med Educ.

[11] Philippine Statistics Authority [Internet]. Results of the 2015 census of population and housing. Manila: Philippine Statistics Authority; 2016. https://psa.gov.ph/population-and-housing/previous-release/2015. Accessed 20 Nov 2019.

[12] Republic of the Philipppines. Republic Act No. 7160: an act providing for a local government code of 1991. Manila: Congress of the Philippines. https://www.lawphil.net/statutes/repacts/ra1991/ra_7160_1991.html. Accessed 20 Nov 2019.

[13] Hyer K, Fairchild S, Abraham I, Mezey M, Fulmer T (2000). Measuring attitudes related to interdisciplinary training: revisiting the Heinemann, Schmitt and Farrell attitudes toward health care teams scale. J Interprof Care.

[14] Parsell G, Bligh J (1999). The development of a questionnaire to assess the readiness of health care students for interprofessional learning (RIPLS). Med Educ.

[15] Tsutusi T, Higashino S (2006). Research related to the status of "collaboration" among municipal public health nurses in Japan. Jpn J Public Health.

[16] Siongco KLL, Nakamura K, Seino K, Moncatar TRT, Tejero LMS, De La Vega SAF (2021). Improving community health workers’ attitudes toward collaborative practice in the care of older adults: an in-service training intervention trial in the Philippines. Int J Environ Res Public Health.

[17] Thorne S (2008). Interpretive description.

[18] Nowell LS, Norris JM, White DE, Moules NJ (2017). Thematic analysis: striving to meet the trustworthiness criteria. Int J Qual Methods.

[19] Reeves S, Lewin S (2004). Interprofessional collaboration in the hospital: strategies and meanings. J Health Serv Res Policy.

[20] Atienza MA, Sana EA (2010). Clinical teaching strategies. Teaching and learning in the health sciences.

[21] Legault F, Humbert J, Amos S, Hogg W, Ward N, Dahrouge S (2012). Difficulties encountered in collaborative care: logistics trumps desire. J Am Board Fam Med.

[22] Haggerty JL, Pineault R, Beaulieu MD, Brunelle Y, Gauthier J, Goulet F (2008). Practice features associated with patient-reported accessibility, continuity, and coordination of primary health care. Ann Fam Med.

[23] Samuelson M, Tedeschi P, Aarendonk D, De La Cuesta C, Groenewegen P (2012). Improving interprofessional collaboration in primary care: position paper of the European Forum for Primary Care. Qual Prim Care.

[24] Sy MP (2017). Filipino therapists’ experiences and attitudes of interprofessional education and collaboration: a cross-sectional survey. J Interprof Care.

[25] Franklin CM, Bernhardt JM, Lopez RP, Long-Middleton ER, Davis S (2015). Interprofessional teamwork and collaboration between community health workers and healthcare teams: an integrative review. Health Serv Res Manag Epidemiol.

