# Supplementary material for: In-service training programme for health and social care workers in the Philippines to strengthen interprofessional collaboration in caring for older adults: a mixed-methods study
Source: Health Res Policy Syst. 2022 Nov 29;20(Suppl 1):111. doi: 10.1186/s12961-022-00914-2 (PMC9706821; doi:10.1186/s12961-022-00914-2)
Supplement: Supplementary file 1 — Additional file 1: Appendix 1. Case studies for training programmes to strengthen interprofessional collaboration in caring for older adults. [file 12961_2022_914_MOESM1_ESM.pdf]

Appendix 1. Case studies for training programmes to strengthen interprofessional collaboration in caring for older adults

Case study 1.

Sheet 1: Description of the barangay, barangay health centre and consultation

Barangay A is situated in an agriculture and tourism focused city, with a potential to develop its agri-based industries such as food processing. The city is rich in natural resources and is a comfortable place to live. It is a calm environment with open grassland and beautiful scenery, despite traffic during rush hour. Most residents use public transportation, such as jeepneys and tricycles. The total population of Barangay A is 7558, with 11% of individuals aged ≥60 years. Most residences are situated in the urban part of town, with a few households living in shanty houses. The Barangay Council is composed of designated leaders for committees on health and education, budget and appropriation, peace and order, sports, infrastructure, disaster and the environment, and a women's group. The barangay is focused on livelihood programmes and strict implementation of appropriate disposal of garbage. They recycle tyres and bottles to create plant and flower pots and have started community vegetable gardens. The barangay hall, which is used by barangay (village) officials to conduct official meetings and gatherings, is located along the highway; the closest private and government hospitals are situated approximately 4 km away, and they are accessible via public and private transportation.

The designated barangay health station is located 1.5 km from the barangay hall. The staff consists of one medical doctor, one public health nurse, one midwife, one barangay nutrition scholar and three barangay health workers. The health station operates Mondays to Fridays, from 08:00 to 17:00, and has a consultation day specifically for senior citizens. Some of the free local health programmes implemented by the health station are a distribution of free medicines each month, such as antihypertensive and antidiabetic agents; free laboratory tests, such as sputum, urine and stool examinations; immunizations and vaccines; and mental health programmes.

Mang Juan, a 68-year-old Filipino male, who lives in Barangay A, went to the health station for a first-time consultation on a Thursday morning with the chief complaint of pain all over his body. Upon entering the health station, Mang Juan and his 30-year-old son were directed to the records section by a health worker. Mang Juan was issued with a new patient health card, and his son completed an assessment form with the following information: the patient's name, address, birth date, age and civil status. Mang Juan and his son were directed to the assessment area where a public health nurse gathered the following information: vital signs (blood pressure, pulse rate, respiratory rate, temperature), height and weight, chief complaint and history of the present illness as well as information about Mang Juan's medical history. Mang Juan had blunted affect. He remained silent most of the time, offering little information about his health condition. He said that he had pain all over his body, but gave only limited details about the character of the pain. A comprehensive geriatric assessment was performed by the public health nurse, and it revealed tenderness over the occiput, scapula, lateral aspect of the elbows and hips, and on both knees. The rest of the physical examination was clinically unremarkable. The patient's blunted affect and complaint of pain prompted the nurse to refer him to the physician for further assessment and evaluation.

Group task 1: List problems and concerns

Sheet 2: Information about family structure, recent life events, history of present illness

Mang Juan is a widower and head of household who lives in a two-storey house made of concrete and light materials in a populated segment of Barangay A with his son, daughter-in-law and 5-year-old grandson. In his early 20s, he migrated from a province in Mindanao for better job opportunities; therefore, there are no other relatives nearby. Mang Juan is known to be a private person, but he enjoys occasional gatherings with his co-workers after work. He is ambulatory and helps care for his grandson by sometimes taking him to school and keeping him company during the weekends. He feels happy sharing time with his son's family and having a home of their own, despite not having many friends and social activities. Mang Juan is a carpenter and earns money on a per-project basis. His son completed a vocational course as an electrician and works on a contractual basis, but he is currently out of work. His daughter-in-law is a stay-at-home mom who provides laundry and ironing services. One month ago, the family lost their house due to a massive fire in their neighbourhood. The family

was relocated to a distant community to start anew. Since then, Mang Juan has been observed to be lost in his thoughts and withdrawn. He is noted to be irritable with his grandson. He has lost interest in carpentry and would rather stay inside the house.

Mang Juan has a history of hypertension and takes one 50 mg tablet of losartan once a day. He was diagnosed with senile cataract and has mild hearing impairment in his left ear. He has no known allergies to medications. He used to experience intermittent pain in both knees many years ago, but can perform activities of daily living independently. He noticed an increase in the frequency and severity of his generalized pain over the past 2 weeks, and this made it difficult to get up from bed and to walk around. He rated the pain as 8/10 in intensity, and as vague, continuous, diffuse and unable to be localized. This pain is accompanied by intermittent headache, dizziness and insomnia. He sought a consultation because of worsening pain, which is accompanied by anorexia and subsequent weight loss.

Upon initial assessment by the public health nurse, his vital signs were: blood pressure – 140/90, pulse rate – 72 bpm, respiratory rate – 18 bpm, and temperature – 36.2 ^{0}C. He weighed 58 kg and his height was 168 cm. He is oriented to date, time and place; has no history of falls and walks with a steady gait and good balance, despite the presence of pain. He can bathe and dress himself, but uses a urinal to avoid going down the stairs to the bathroom. He used to help clean the house, but now he prefers to stay in his room most of the time and watch TV. His daughter-in-law prepares the meals for the family. Bladder and bowel function are unremarkable. He has not undergone any major or minor surgery, nor has he been admitted to hospital for any illness.

During the consultation with the doctor, Mang Juan said, “I feel some pain in my body”. His son expressed concern regarding Mang Juan’s change in mood, loss of appetite and being quiet most of the time. In addition, the son also mentioned that “It looks like my father lost the will to live. He seems to be getting weaker by the day. I am worried about his health”.

Due to his present condition, Mang Juan’s daily activities are significantly decreased: he usually stays upstairs because of difficulty in walking down a flight of 10 steps due to pain, chooses to watch TV rather than play with his grandson and has no social interactions outside of those with his family.

The doctor referred Mang Juan to a geriatrician in a hospital. During the consultation, the geriatrician performed a comprehensive geriatric assessment. Hospital admission was advised for further assessment, but the family refused due to financial considerations. So, the geriatrician provided prescriptions for laboratory examinations, pain medications and antihypertensive agents, physical therapy and mental health counselling.

Prior to the fire, Mang Juan used to receive free medicines at the health station, which was only 400 m from his home. After being relocated, Mang Juan did not have enough savings to cover rent and basic needs, such as food and clothing. What little savings his son has is used for his own son’s education and can cover food and utility expenses, but this is not always possible due to irregular work.

Group task 2: Propose what the team should do next
Group task 3: Apply the comprehensive geriatric assessment tool and list problems and concerns

Sheet 3: Epilogue – successful outcomes

The interprofessional team consists of the health station doctor, the public health nurse, the health worker, the physical therapist or occupational therapist and a social worker. A comprehensive assessment of Mang Juan was carried out, with assistance in record management by the health worker, the initial medical and family history taken by the public health nurse, and the medical consultation carried out by the doctor. A mental health assessment was done by collecting information on the present illness and using relevant assessment tools (e.g., a geriatric depression scale). Gathering the relevant medical and social histories enabled the health worker, public health nurse and doctor to determine whether there was a need for regular counselling and treatment of comorbidities, such as hypertension and generalized pain. The doctor determined the required medical treatment and evaluated Mang Juan’s living conditions and social support; the public health nurse evaluated his needs for nursing care and ability to carry out activities of daily living, and gave instructions about the

prescribed medications and the importance of medication compliance; the nutrition scholar evaluated whether the patient ate a balanced diet and monitored his weight; the physical therapist focused on improving the functional levels in the physical domains of mobility, strength, sensation and pain; and the social worker made the necessary arrangements for the family to facilitate home care for the patient. In addition, the social worker was also on hand to evaluate the needs of the patient, which led to engagement with programmes of social activities for older people and programmes offering financial assistance to cover the costs of health care and daily needs. By identifying community-based resources, the social worker was also able to assist the son in finding a job. The interprofessional team has the capacity to allow the patient to be cared for at home as a result of performing a comprehensive assessment and making adjustments as necessary.

Because the family cannot afford to travel to the health station, the health worker and public health nurse make regular home visits to provide monthly maintenance medications and counselling and to follow up on his medical and mental health conditions. The interprofessional team focused on relieving Mang Juan's symptoms and helping him return to his daily routine.

By holding regular team meetings and maintaining an open line of communication between team members, the team can develop and evaluate therapeutic strategies consistent with the patient's rehabilitation goals. Within a month of the initial consultation, the observed changes included a reduction in pain, increased weight and a return to independent living. The patient's self-image had improved, and he has slowly regained control over his life. He began voluntarily participating in social activities within the barangay, improved communication with his family members and health workers, and is helping around the house by using his carpentry skills.

Finances remain a problem for the household, but the patient's current condition has not created a further burden on their savings because of the efficient care provided by the team. The patient also currently receives a social pension as a result of assistance in enrolment provided by the social worker. The care plan is updated whenever the public health nurse reports on his current medical and mental health status, the physical therapist reports on his functional capacity and the social worker reports on his social engagement to reflect the medical, nursing and social interventions needed to further improve the patient's overall well-being. Interprofessional collaboration among the health and social care professionals prevented further decline in the patient's physiological status and psychological status that could have led to hospital admission and inevitable and catastrophic household expenditure for medical treatment.

Sheet 4: Epilogue – unsuccessful outcomes

In the absence of intervention, the signs that Mang Juan's pain could be contributing to his psychological distress included:

1. withdrawing from pleasurable activities due to fear of having an accident;
2. losing his appetite or decreasing his fluid intake dramatically, or both;
3. asserting feelings of sadness, helplessness or worthlessness, or some combination of these;
4. changing sleep patterns;
5. increasing irritability, agitation or anger, or some combination of these;
6. scheduling multiple appointments with the physician for the same complaint;
7. diminishing quality of work life.

In the case of unsuccessful outcomes, the family will likely have catastrophic out-of-pocket expenditures for medical treatment and possibly for hospital admission caused by further declines in his health.

Case study 2.

Sheet 1: Description of the barangay, barangay health centre and consultation

Barangay B is situated in a highly urbanized city, with a total population of more than 450 000, and it has several commercial and industrial centres, as well as many markets in all parts of the city. The city is located along the eastern border of the province and is surrounded by lush greenery and a river that runs to the western section of the city. During the rainy season, the river can rise to 15 m. The city is an ideal residential area for workers in the province, and this has resulted in the development of several subdivisions and government housing villages, as well as many households living in clusters of shanty houses. Most residents use public transportation, such as taxis, jeepneys and tricycles. Roads are narrow, and the inflow and outflow of people contribute to heavy traffic during rush hour. The total population of Barangay B is 20 558, with 4.5% of individuals aged ≥60 years. The barangay hall, which is used by barangay (village) officials to conduct official meetings and gatherings, is located along the highway.

The designated barangay health station is adjacent to the barangay hall. The staff consists of one medical doctor, one public health nurse, two midwives, one nutritionist, one barangay nutrition scholar and four barangay health workers. The health station operates Mondays to Fridays, from 08:00 to 17:00, and has a consultation day specifically for senior citizens. Some of the free local health programmes implemented by the health station for older people are home visiting; a distribution of free medicines each month, such as antihypertensive and antidiabetic agents; free laboratory tests, such as sputum, urine and stool examinations; and immunizations and vaccines. The local government unit office, through its social welfare office, also provides social pensions and capacity building for the elderly population.

The city has several private hospitals and one government tertiary referral hospital, all of which are easily accessible. The main tertiary referral facility is a teaching and training hospital, and it cares for complicated cases in the province, as well as from nearby provinces. It has a 300-bed capacity and admits cases needing medical; surgical; obstetric-gynaecological; paediatric; ear, nose and throat/ophthalmology; and orthopaedic care. It is supported by different cadres of health professionals, including medical consultants, medical residents, nurses, allied health professionals (e.g., physical therapists, medical technologists, pharmacists, nutritionists, medical social service workers, health information specialists), dentists, midwives and nurses' aides. The annual hospital occupancy rate is above 100%, given that it is the lone government referral hospital providing services and it welcomes patients from all walks of life.

Aling Meding, a 62-year-old Filipina female, who resides in the barangay, was transported to the emergency room at the government tertiary hospital due to sudden onset of weakness in both her right arm and right leg at 05:00, while she was preparing to go to work as a vendor at the nearby community market. Despite the weakness, she decided to continue with her preparations for work. But at 05:20, she noticed that the weakness made her unable to carry a bag and walk unassisted. Her daughter rushed to the barangay hall to request transport assistance to the hospital. During transit to the hospital, she developed right facial asymmetry and slurring of speech. Aling Meding and her daughter arrived at the emergency room at 06:45, and she was moved to the critical care unit of the emergency room via wheelchair. The emergency room nurse took her vital signs: blood pressure – 160/80; cardiac rate – 96 (regular rhythm); respiratory rate – 20; and temperature – 36.7 ^{o}C. The daughter described the series of events because Aling Meding was unable to speak clearly due to slurred speech. The nurse suspected onset of acute stroke and immediately referred the patient to a neurology resident.

Group task 1: List problems and concerns

Sheet 2: Information about family structure, recent life events, history of present illness

Aling Meding is a widow who lives 1 km from the barangay health station. Her house is one-storey and made of concrete and light materials; it is situated in a densely populated area of the barangay. Her only daughter – who is not married and works as a cashier in one of the grocery stores near the community market – lives with her but is not able to regularly attend to her health care needs because of her full-time job. Aling Meding does not smoke or drink alcoholic beverages. She works as a vendor

in the community market: her employer provides the vegetables and goods to be sold. Aling Meding is covered by mandatory national health insurance with the Philippine Health Insurance Corporation (PhilHealth), which covers medical care for seniors in public hospitals; she has a social pension; and she uses her senior citizen's card to obtain a 20% discount and VAT exemption on applicable goods and services. Mother and daughter are barely surviving financially.

Aling Meding was seen by the neurology resident at 07:00. Neurological examination revealed a drowsy patient who was able to follow simple commands and who had incomprehensible verbal output. She had facial asymmetry on the right. Manual muscle testing revealed 0/5 muscle strength on the right and 3/5 on the left, in both the upper and lower extremities. The neurology resident ordered 1 L of intravenous plain normal saline at 80 mL/hour and a single dose of 40 mg of atorvastatin. This was followed by scheduling an appointment for plain magnetic resonance imaging (MRI), which was done at 07:45 and revealed an acute cerebral infarct, left. This was the first documented occurrence of symptoms, and because Aling Meding was within the golden time of 3 to 4.5 hours from the onset of symptoms, recombinant tissue plasminogen activator (rtPA) was administered, which required intensive monitoring.

Her medical history revealed that she has had hypertension and diabetes mellitus for the past 20 years. She takes 50 mg of losartan twice a day and 500 mg of metformin twice a day after meals. She visits the health station regularly for follow up and monitoring of her laboratory parameters. She also receives her maintenance medicines from the health station.

Her daughter is worried about the financial burden they will incur for the costs of medicines, procedures, the hospital stay and rehabilitation. Despite having PhilHealth insurance, most medicines and some of the laboratory exams will not be covered.

Aling Meding was admitted to the intensive care unit for 4 days for prompt attention from physicians and nurses to prevent further complications. In addition to acute stroke management, nasogastric tube feeding was started because she had difficulty swallowing. Blood pressure and blood sugar control were instituted. No further complications developed, as evidenced by the results of inpatient laboratory exams; thus, she was cleared by the doctor for transfer to a general medical–surgical ward on the fifth day post-stroke.

In the general medical–surgical ward, the cardiologist focused on controlling her blood pressure, and the neurologist ensured there was adequate brain reperfusion following the stroke. The endocrinologist helped her maintain normal blood sugar levels while coordinating with the dietitian for nutrition counselling.

Upon transfer to the general ward, Aling Meding was referred to a physiatrist so that physical and speech rehabilitation therapy could be started; she was also referred to a social worker.

Her daughter was usually outside the hospital looking for medical assistance and seeking additional funds. Aling Meding was discharged after 2 weeks and was instructed to continue her medications and to have rehabilitation sessions three times per week in the tertiary hospital, but she and her daughter were contemplating seeking the services of a traditional hilot practitioner instead. Upon returning home, the daughter went back to work immediately to earn money, which meant that Aling Meding was left alone at home. Purchasing medications seemed to be a problem. The daughter decided to visit the health station to seek assistance in providing and sustaining medical and social care for her mother.

Group task 2: Propose what the team should do next
Group task 3: Apply the comprehensive geriatric assessment tool and list problems and concerns

Sheet 3: Epilogue – successful outcomes

The immediate provision of transport by the barangay council facilitated Aling Meding's timely arrival to the emergency room and, consequently, early recognition of stroke symptoms within the golden time, which led to appropriate interventions that prevented further complications. A comprehensive geriatric assessment was carried out by the nurse and physician in the emergency room to further investigate what might have caused her symptoms. This prompt and accurate assessment led to the

initiation of rtPA therapy, which significantly reduced her symptoms. Blood tests, an electrocardiogram and an MRI were ordered by the physician and carried out by the nurse within 24 hours of admission to the emergency room.

The hospital's interprofessional team is composed of doctors, nurses, nurses' aides, rehabilitation therapists and social workers. Admission to the critical care unit for intensive monitoring prevented potential harmful side effects of rtPA therapy. Continual treatment, with regular monitoring and assessments, were provided by the doctors and nurses, as well as the nurses' aides. The doctor, nurse and nurses' aides were in constant communication, thus ensuring that care was delivered effectively and efficiently. The patient was also seen by doctors specializing in stroke for further assessment to prevent life-threatening infections and to reduce the chance that she would develop complications.

Because there were no further complications, the patient was transferred from the intensive care unit to the general medical–surgical ward and received routine assessment and management. She was also referred to the rehabilitation unit, the staff of which is composed of doctors, and to physical, occupational and speech therapists, for evaluation and treatment. Integrated care and rehabilitation goals were achieved by conducting interprofessional meetings attended by the cardiologist, neurologist, nurses, physical and speech therapists, and nurses' aides. The meetings provided an opportunity for all professionals to receive timely updates about the patient's condition, to address polypharmacy and to make careful decisions about the laboratory exams to be ordered to prevent further financial burden. The referral to social work services helped the daughter to receive funds from the government to cover the costs of medical expenses.

Prior to discharge, Aling Meding and her daughter were taught by the health care team about procedures to ensure safety and to meet their physical and emotional needs, which allowed Aling Meding to recover at home and would also help to prevent irreversible damage. Including the social care worker on the team before discharge ensured that links were provided to other financial support mechanisms offered by local and national governments to ease the catastrophic financial effects.

Referrals from the hospital to the health station resulted in continuity of care and the patient's use of free services, such as receiving free maintenance medications, laboratory exams and physical therapy sessions. Home visits were performed by the barangay health workers, nurses and physicians to follow up on the patient's condition. Clinical improvement was progressively observed from admission until discharge. A focus on exercises to regain fitness and muscle strength, walking and balance and gait training allowed the patient to return to independent functioning.

The patient's overall self-image and self-worth improved, and she returned to work after several months. Out-of-pocket payments for medical needs were significantly reduced. Interprofessional collaboration among the health and social care professionals in the hospital during admission and referral to the community health care team prevented further decline in physiological, functional and psychological status that could have led to hospital readmission and catastrophic out-of-pocket expenditures.

Sheet 4: Epilogue – unsuccessful outcomes

If Aling Meding did not receive timely and integrated care, she may have had the following results.

1. Late management will lead to irreversible effects of stroke (such as permanent muscle weakness, facial asymmetry, slurred speech).
    - She might have problems understanding speech or speaking.
    - She might have problems swallowing.
    - She might have problems seeing.
    - She might have difficulties with balance or coordination.
    - She may become depressed.
    - She may experience numbness or strange sensations.
    - She may have pain in her hands and feet that worsens with movement and temperature change.
2. She may have difficulties thinking, or in awareness, attention, learning, judgement and memory.
3. She may develop one of the potential medical complications of stroke, including:
    - myocardial infarction or heart failure;

- deep vein thrombosis or a pulmonary embolism;
- infections or pneumonia;
- pressure sores;
- malnutrition and dehydration;
- dementia.

4. Her quality of health and life may decline.
5. She may be unable to regain independent functioning and so lose her job.
6. She may become a burden to her caregiver.

Unsuccessful outcomes will lead to a financial burden that the household cannot sustain; they will also lead to a poor prognosis, which will eventually lead to premature death.
